# Protective effects of curcumin against osteoporosis and its molecular mechanisms: a recent review in preclinical trials

**DOI:** 10.3389/fphar.2023.1249418

**Published:** 2023-09-18

**Authors:** Shenglei Yang, Yuying Sun, Leonid Kapilevich, Xin’an Zhang, Yue Huang

**Affiliations:** ^1^ College of Exercise and Health, Shenyang Sport University, Shenyang, China; ^2^ School of Stomatology, Binzhou Medical College, Yantai, China; ^3^ Faculty of Physical Education, Nаtionаl Reseаrch Tomsk Stаte University, Tomsk, Russiа

**Keywords:** osteoporosis, curcumin, osteoblast, osteoclast, mechanism

## Abstract

Osteoporosis (OP) is one of the most common metabolic skeletal disorders and is commonly seen in the elderly population and postmenopausal women. It is mainly associated with progressive loss of bone mineral density, persistent deterioration of bone microarchitecture, and increased fracture risk. To date, drug therapy is the primary method used to prevent and treat osteoporosis. However, long-term drug therapy inevitably leads to drug resistance and specific side effects. Therefore, researchers are constantly searching for new monomer compounds from natural plants. As a candidate for the treatment of osteoporosis, curcumin (CUR) is a natural phenolic compound with various pharmacological and biological activities, including antioxidant, anti-apoptotic, and anti-inflammatory. This compound has gained research attention for maintaining bone health in various osteoporosis models. We reviewed preclinical and clinical studies of curcumin in preventing and alleviating osteoporosis. These results suggest that if subjected to rigorous pharmacological and clinical trials, naturally-derived curcumin could be used as a complementary and alternative medicine for the treatment of osteoporosis by targeting osteoporosis-related mechanistic pathways. This review summarizes the mechanisms of action and potential therapeutic applications of curcumin in the prevention and mitigation of osteoporosis and provides reference for further research and development of curcumin.

## 1 Introduction

Osteoporosis (OP) is a metabolic bone disease commonly occurring in the elderly and can lead to progressive loss of bone mineral density, persistent deterioration of bone microarchitecture, and increased fracture risk (Lee and Choi, 2011; Rachner et al., 2011). OP can affect men and women, and women are more likely to develop severe osteoporotic complications (Kaunitz et al., 2009). The emergence of OP may be associated with various factors, including aging, estrogen deficiency, and reduced mechanical stimulation (Johnston and Dagar, 2020; Cheng et al., 2022; Wang et al., 2022). More than 200 million people worldwide have OP, and its prevalence may be higher in countries and regions with more severe population aging (Ballane et al., 2017; Clynes et al., 2020). Currently, the primary treatment modalities for OP include medication and preventive measures to improve patients’ quality of life (Khosla and Hofbauer, 2017). Existing treatments, such as estrogen replacement therapy (ERT), bisphosphonates, parathyroid hormone (PTH), and calcium supplements, can alleviate the progression of OP. However, most of these drugs are expensive and have long treatment cycles, which may lead to serious side effects (Ruggiero and Likis, 2002; van der Mooren and Kenemans, 2004; Sugiyama et al., 2015; Reid and Billington, 2022). Therefore, novel effective therapies for OP must be developed.

Natural plant active substances can be widely used as an alternative to prevent and treat OP because of their extensive biological activities (Martiniakova et al., 2020; Yang et al., 2022). Compared with chemically synthesized drugs, herbal medicines are preferred by researchers due to their wide distribution, low cost, and few adverse effects. Curcumin (CUR), derived from turmeric, is a naturally occurring polyphenolic compound (Kocaadam and Şanlier, 2017). CUR has a wide range of biological activities and is used to treat various diseases, such as cancer, diabetes, cardiovascular diseases, and other inflammatory diseases (Hong et al., 2019; Abu-Taweel et al., 2020; Pourbagher-Shahri et al., 2021). Furthermore, studies on the beneficial aspects of CUR on bones have been widely published (Zhou et al., 2013; Kunihiro et al., 2020). Moreover, CUR treatment significantly improved bone density and bone microarchitecture in APP/PSI transgenic mice (Kunihiro et al., 2020). CUR is expected to be a safe and effective therapeutic agent for OP given its remarkable properties in disease prevention and treatment and has received increasing attention from researchers in the past decade. However, studies on the effects of CUR on OP remain limited. We hypothesize that CUR may be a pleiotropic molecule targeting OP with various biological activities. This review aims to describe the effects of CUR on OP and discuss the molecular mechanisms by which it alleviates OP to provide clinical implication.

## 2 Basic overview of curcumin

**FIGURE 1:**
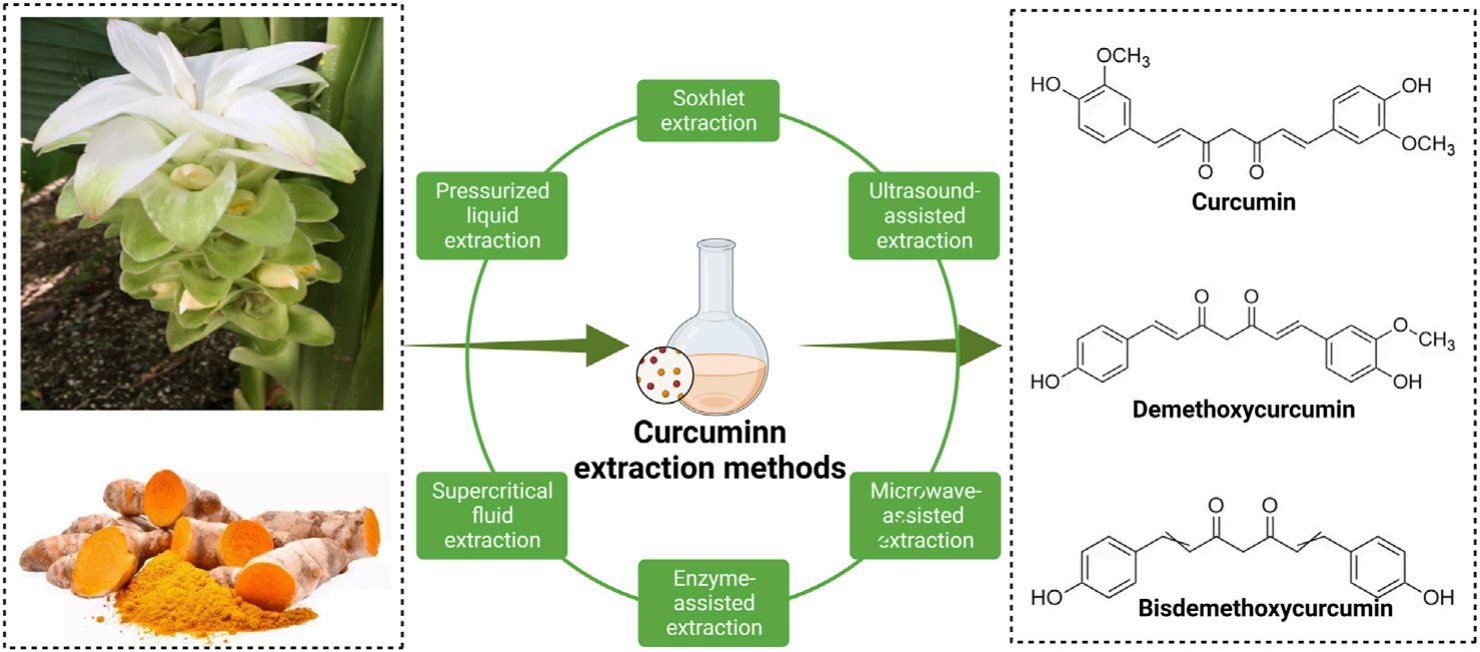
Three main chemical structures of curcumin compounds and the main methods of extracting curcumin from plants.

Turmeric (*Curcuma longa*) is a herb known as golden spice. The root parts of turmeric are widely used not only as a dietary ingredient but also as a traditional medicine to treat various diseases; they play a pivotal role in Ayurvedic medicine and herbalism (Deogade and Ghate, 2015; Nelson et al., 2017). CUR-like compounds are essential natural polyphenols extracted from the rhizome parts of turmeric and mainly include curcumin (77%), dimethoxy curcumin (17%), and bisdemethoxycurcumin (3%) (Goel et al., 2008) (Figure 1). CUR is the most bioactive chemical substance in turmeric, and it exists in the form of keto-enol and exhibits different activities in acidic and alkaline solutions (Manolova et al., 2014).

CUR has been widely used to prevent and treat various diseases due to its broad pharmacological activity and multimolecular targeting effects. It is a well-tolerated and safe human compound through clinical trials (Shishodia et al., 2005)CUR has received increasing attention from researchers. Although CUR has beneficial effects, its low solubility, rapid metabolism, low stability, and poor absorption and bioavailability in living organisms greatly limit its application as a bioactive supplement (Devassy et al., 2015; de Souza Ferreira and Bruschi, 2019). Researchers have improved the study of CUR applications *in vitro* and *in vivo* by proposing a nanotechnology-based strategy to address these issues. Nanoparticles, such as micelles, liposomes, and nanogels, enhance the efficiency of CUR by increasing its solubility and blood circulation time and blocking the metabolic pathway to increase bioavailability (Ghalandarlaki et al., 2014).

In the past, CUR was used in Ayurvedic medicine in India to treat infections, skin diseases, and acne (Gupta et al., 2013; Deogade and Ghate, 2015). n modern times, as researchers continue to explore the biological activity of CUR, it has been proven by the US Food and Drug Administration (FDA) to be a natural compound that exerts a variety of pharmacological activities, such as antioxidant, anti-apoptotic, and anti-inflammatory (Patil et al., 2009; Hewlings and Kalman, 2017). CUR exerts its pharmacological activity by targeting various cells and modulating different signaling pathways. CUR plays an essential regulatory role in developing several diseases by modulating mitochondrial function and altering various cytokines, transcription factors, kinases, and apoptotic molecules (Shehzad and Lee, 2010; Kooti et al., 2017; Gorabi et al., 2019). Previous studies showed that elevated reactive oxygen species (ROS) caused by mitochondrial dysfunction affect osteoblasts by regulating apoptosis and mitochondrial DNA damage (She et al., 2014; Yang et al., 2014). CUR may effectively prevent osteoblast dysfunction by improving mitochondrial function (Dai et al., 2017). Moreover, CUR promotes the differentiation of rat bone marrow mesenchymal stem cells to osteoblasts by upregulating the heme oxygenase-1 (HO-1) pathway (Gu et al., 2012).

## 3 Effect of curcumin on bone remodeling

**FIGURE 2:**
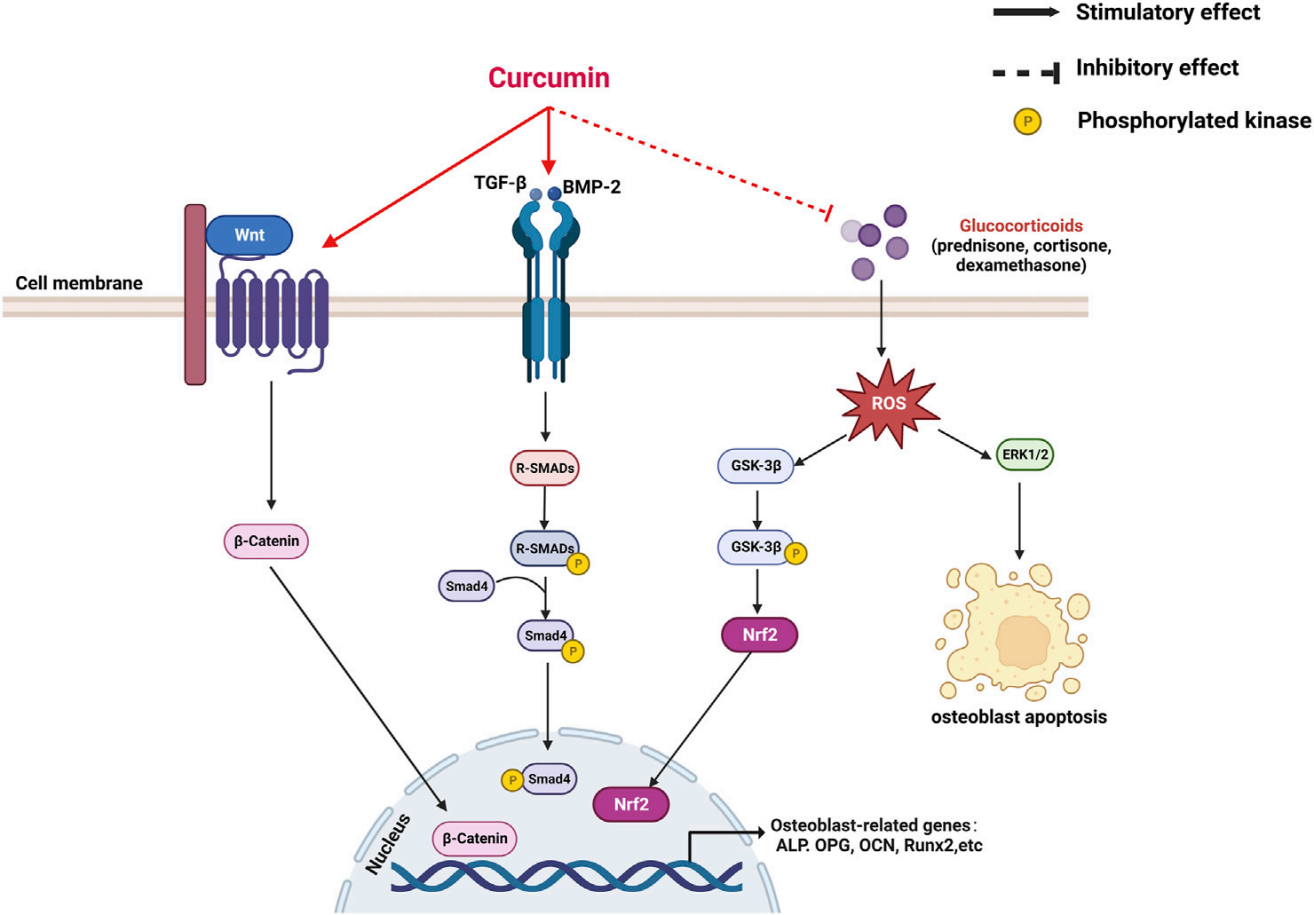
A mechanistic model for the promotion of curcumin on osteogenesis.

**FIGURE 3:**
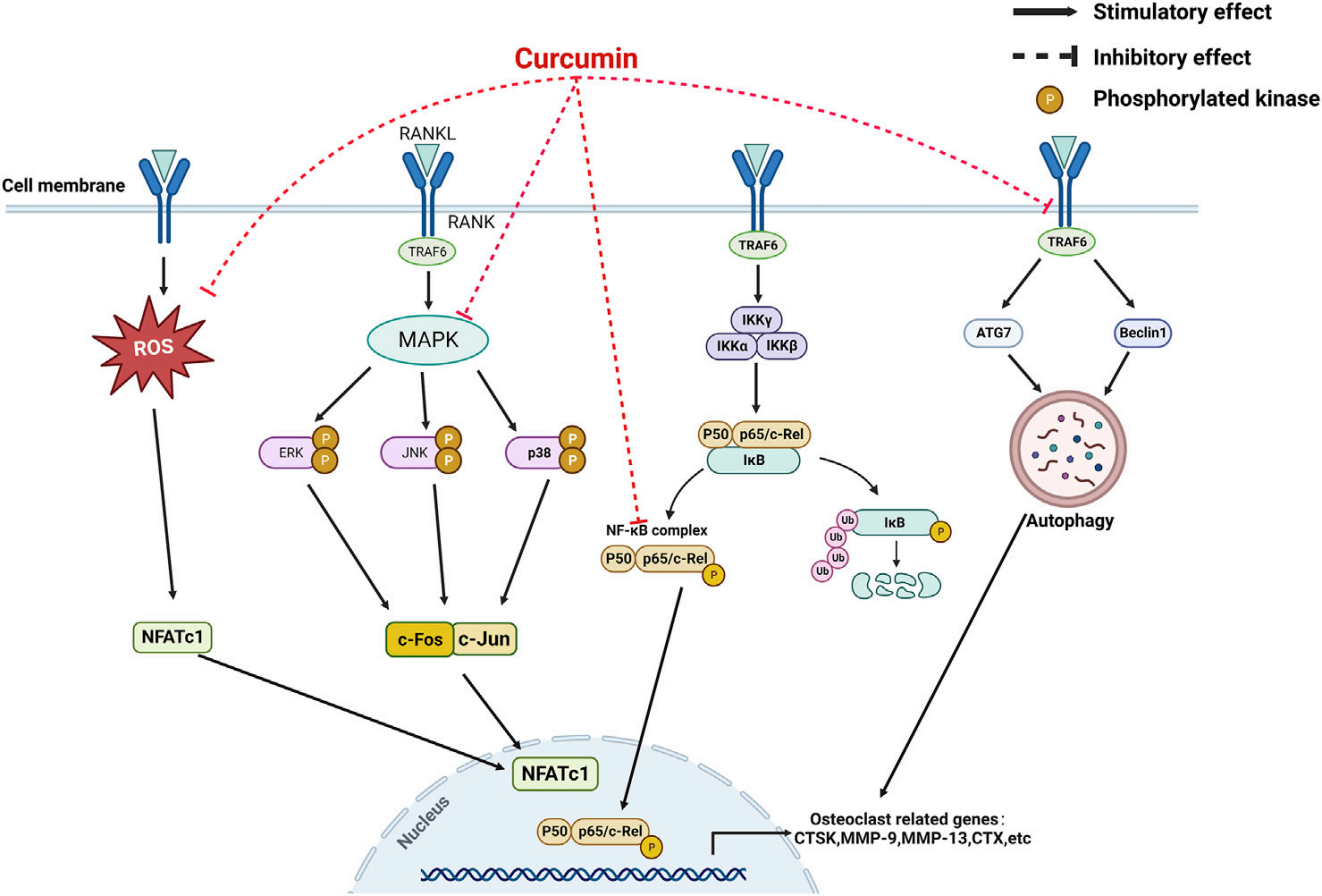
A mechanistic model for the inhibition of curcumin on osteoclastogenesis.

Bone remodeling is an ongoing process, in which a mature bone tissue is disintegrated (known as bone resorption) and replaced by a new bone tissue (known as bone formation) (Miao et al., 2011). The dynamic balance between the two processes is essential for proper bone metabolism. OP is triggered when this equilibrium is disrupted (Shi et al., 2014). Osteoblasts, which mediate bone formation, and osteoclasts, which mediate bone resorption, are key cell lines in bone remodeling; the apoptosis of osteoblasts and the increased activity of osteoclasts are the main pathogenic mechanisms of OP (Guañabens et al., 2014). An increasing number of studies have demonstrated the protective role of CUR in bone health (Inchingolo et al., 2022). CUR can promote osteoblast differentiation and inhibit osteoclast formation from maintaining bone health. This review describes the effects of CUR on osteoblasts and osteoclasts and their molecular mechanisms (Table 1) (Figures 2, 3).

**TABLE 1:** The role of curcumin in bone remodeling.

Cells model	Dosage range	Active concentration	Functions	Signaling pathways/Mechanisms	References
Curcumin promotes osteogenic differentiation					
*In vitro*, MC3T3-E1 cells	1–2000 nM	10 nM	Promotes osteoblasts differentiation, proliferation and mineralization	Wnt/β-catenin and Smad signaling pathways	Yamaguchi et al. (2012), Li et al. (2015), Chen et al. (2016b), Bukhari et al. (2019)
*In vitro*, primary osteoblasts	0.5, 1 and 2 μM	0.5 μM			
*In vitro*, MC3T3-E1 cells	4 μM	4 μM	Protects osteoblasts from oxidative stress and apoptosis-induced dysfunction	GSK3β-Nrf2 and ERK signaling pathways	Xin et al. (2015), Chen et al. (2016a), Li et al. (2020)
*In vitro*, primary osteoblasts	0.5, 1 and 2 μM	0.5 μM		Inhibits the formation of pro-apoptotic proteins and promotes the formation of anti-apoptotic proteins	
				Inhibits oxidative stress	
Curcumin inhibits osteoclast formation					
*In vitro*, BMMs induced by RANKL	0–25 μM	5 μM	Inhibits osteoclasts differentiation and formation	Akt/NF-κB/NFATc1 and NF-κB signaling pathways	Oh et al. (2008), Hie et al. (2009), Kim et al. (2011), Park et al. (2011), Yamaguchi et al. (2012), Li et al. (2015), Bukhari et al. (2019), Liang et al. (2020b), Yang et al. (2020)
*In vitro*, RAW 264.7 macrophages induced by RANKL	1–10 μM	1 μM			
*In vitro*, RAW 264.7 macrophages induced by RANKL	4 μM	4 μM	Prevents osteoclasts formation by reducing oxidative stress	Suppresses MAPK/NFATc1 signaling pathways	Kim et al. (2011), Xin et al. (2015)
				Inhibits oxidative stress	
*In vitro*, BMMs induced by RANKL	0.4 μM	0.4 μM	Prevents osteoclasts formation by inhibiting autophagy	Promotes autophagy activity	Ke et al. (2020)

Abbreviations: Wnt, wingless and int-1; Smad, recombinant mothers against decapentaplegic homolog; GSK-3β, glycogen synthase 3β; Nrf2, nuclear factor-like 2; ERK, extracellular regulated protein kinases; BMMs, bone marrow-derived macrophages; RANKL, nuclear factor receptor activator kappa B ligand; Akt, protein kinase B; NF-κB, nuclear transcription factor-κB; NFATc1, nuclear factor of activated T cells 1; MAPK, mitogen-activated protein kinase pass.

### 3.1 Mechanism of anti-osteoporotic effect of curcumin on osteoblasts

Osteoblasts originate from mesenchymal stem cells (MSCs), which play a crucial role in bone development and formation (Hojo and Ohba, 2020). MSCs differentiate into osteoblasts, chondrocytes, or adipocytes in response to transcription factors. Runx-related transcription factor 2 (Runx2) is a crucial transcription factor for the differentiation of MSCs and pre-osteoblasts into osteoblasts with many targets and downstream regulators, including osterix (Osx) and Sp7(Komori, 2019). Runx2 also regulates the secretion of bone matrix proteins, such as osteocalcin (OCN) and type I collagen (COL1A1) (Ducy et al., 1997). A large body of evidence suggests that the apoptosis of osteoblasts under pathological conditions leads to an imbalance in bone formation–resorption, which is closely associated with the development of OP (Komori, 2016; Chen et al., 2018). Therefore, effective delay of osteoblast apoptosis or promoting osteoblast differentiation may be promising in preventing and treating OP.

CUR plays a role in bone formation by regulating the differentiation of osteoblasts. CUR promotes osteoblast proliferation and upregulates the expression of genes related to bone formation, including alkaline phosphatase (ALP), OCN, and Runx2 (Li et al., 2015; Bukhari et al., 2019). Notably, CUR combined with chasteberry (FLL) effectively promoted osteoblast-mediated bone formation (Bukhari et al., 2019). Furthermore, the knockdown of the β-catenin gene in mice revealed the inhibition of osteoblast differentiation while promoting the differentiation of MSCs toward adipocytes. This finding suggests that the wingless and int-1(Wnt)/β-catenin pathway is an essential regulatory pathway in bone formation (Song et al., 2012). Chen et al. demonstrated that CUR mainly upregulated the phosphorylation activity of glycogen synthase 3β (Gsk3β) to promote the nuclear translocation of β-catenin and thus exert osteoprotective effects (Chen et al., 2016b). Researchers are interested in CUR analogs because of their role in OP. Mawani et al. identified three CUR analogs with potential anti-osteoporotic activity in MG-63 cells (Mawani and Orvig, 2014). A novel CUR analog, UBS109, upregulated the expression of osteogenic-related genes in MC3T3-E1 cells and promoted the bone formation and mineralization in osteoblasts by activating Smad; hence, its chemical structure is associated with osteogenic differentiation (Yamaguchi et al., 2012).

CUR is a bioactive antioxidant that can efficiently scavenge intracellular ROS and free radical activity; as such, it is thought to be beneficial in improving and preventing OP (Pulido-Moran et al., 2016). Evidence suggests that oxidative stress (OS)-induced apoptosis of osteoblasts plays a crucial role in the pathogenesis of OP (Manolagas, 2010). Xin et al. placed MC3T3-E1 cells in a rotating wall vascular bioreactor (RWVB) to simulate a microgravity environment *in vitro*. They found that long-term exposure to microgravity increased the level of oxidative stress and promoted the increase in ROS content in cells. After CUR treatment, the ROS expression decreased and osteoblast differentiation was promoted (Xin et al., 2015). Gsk3β is a serine/threonine kinase involved in bone metabolism and mediates OS-induced apoptosis in osteoblasts by promoting the release of cytochrome c from mitochondria (Leevers et al., 1999; Mudher et al., 2001). Dai et al. found that CUR attenuates OS-induced apoptosis in osteoblasts by activating protein kinase B (Akt) to inactivate downstream Gsk3β phosphorylation (Dai et al., 2017). In addition, Gsk3β downregulates the expression of nuclear factor-like 2 (Nrf2), a key transcription factor for cellular antioxidant defense involved in apoptosis (Zhuang et al., 2019). Nrf2 has been suggested as a potential target to slow down the progression of skeletal degenerative diseases (Jiang et al., 2018; Olagnier et al., 2018). Li et al. demonstrated that CUR rescued mouse MC3T3-E1 pre-osteoblasts from damages caused by OS by inhibiting the Gsk3β/Nrf2 signaling pathway (Li et al., 2020). In an *in vitro* experiment, Chen et al. exposed osteoblasts to dexamethasone (Dex); the process induced massive apoptosis accompanied by a decrease in ERK phosphorylation levels. CUR treatment prevented Dex-induced OP by inhibiting osteoblast apoptosis and upregulating the extracellular regulated protein kinase (ERK) phosphorylation (Chen et al., 2016a). In conclusion, CUR can exert osteoprotective effects by promoting osteoblast differentiation and inhibiting apoptosis.

### 3.2 Mechanism of anti-osteoporotic effect of curcumin on osteoclasts

Osteoclasts originate from the monocyte-macrophage lineage and are the primary functional cells of bone resorption; they can break down bone minerals and degrade the organic bone matrix (Suda et al., 1999). In general, the differentiation of macrophages into osteoclasts depends on the secretion of bone marrow stromal cells (BMSCs) or osteoblasts to release signaling factors necessary for osteoclast formation; these factors include macrophage colony-stimulating factor (M-CSF) or nuclear factor receptor activator kappa B ligand (RANKL) (Pereira et al., 2018). RANKL is a crucial regulator of osteoclast differentiation and is expressed by bone marrow stromal cells, osteoblasts, and osteoclasts (Kim et al., 2020). It binds to the receptor activator of nuclear factor kappa B (RANK) on the membrane of osteoclast precursor cells and activates the corresponding pathways of osteoclastogenesis, such as mitogen-activated protein kinase pass (MAPK), protein kinase B (Akt), and nuclear transcription factor-κB (NF-κB); RANKL promotes the expression of other osteoclastic factors, such as a nuclear factor of activated T cells 1 (NFATc1) and c-fos (Takayanagi, 2005; Park et al., 2017). The enhanced osteoclast overactivity under pathological conditions, such as inflammatory stimuli, decreased the bone reduction in volume, resulting in OP(Locantore et al., 2020; Ratajczak et al., 2020). Therefore, inhibition of osteoclast activity and its related signaling pathways that mediate bone resorption has become potential therapeutic targets for the prevention and treatment of OP.

CUR plays a role in bone resorption by regulating the differentiation of osteoclasts. Bukhari et al. found that CUR combined with FLL significantly inhibited the proliferative activity of RAW264.7 cells (Bukhari et al., 2019). In addition, CUR blocked the differentiation of osteoclast precursors into osteoclasts by inhibiting the production of chemokine CCL3 (Liang Z. et al., 2020). Similarly, CUR ameliorated microgravity-induced bone loss by inhibiting reactive oxygen species production and decreasing osteoclast-related gene expression (Xin et al., 2015). Micro RNAs develop OP by mediating osteoclast-regulated bone resorption (Lee et al., 2021). Li et al. found that CUR inhibited osteoclast differentiation markers, including cathepsin K, matrix metalloprotein (MMP)-9, and MMP-13, by upregulating the expression of miR-365 (Li et al., 2015). RANKL also plays a crucial role in osteoclast differentiation and activation. RANKL knockout mice prevented bone loss during arthritis, suggesting that osteoclast bone resorption under inflammatory conditions depends on RANKL/RANK signaling (Zeng et al., 2020; Xia et al., 2022). Hence, the RANKL/RANK pathway is a key pathway for osteoclast formation and is expected to be a potential target for the treatment of OP. Oh et al. demonstrated that CUR treatment in a co-culture system reduced bone loss caused by increased osteoclasts by inhibiting interleukin-α (IL-1α)-induced RANKL expression in bone marrow mesenchymal stem cells (Oh et al., 2008). Similarly, Park et al. found a robust inhibitory effect of CUR analogs on RANKL-induced osteoclastogenesis by assaying tartaric acid-resistant acid phosphatase (TRAP) activity in mouse RAW264.7 cells (Hie et al., 2009; Park et al., 2011). Similarly, Yamaguchi et al. demonstrated that CUR analogs (ECMN909 and UBS109) significantly inhibited RANKL-induced NF-κB activity in osteoblast precursor RAW264.7 cells and blocked their differentiation into osteoclasts *in vitro* (Yamaguchi et al., 2012). However, whether autophagy can promote osteoclastogenesis remains controversial; CUR, an autophagy activator and osteoclastogenesis inhibitor, inhibits osteoclast activity. For the first time, Ke et al. explained the underlying mechanism behind this paradoxical effect. They confirmed that CUR prevented bone loss by inhibiting the stimulatory effect of RANKL on osteoclast autophagy (Ke et al., 2020).

As a multi-targeted polyphenol, CUR exerts osteoprotective effects by modulating osteoclast-mediated bone resorption-related pathways. The MAPK and NF-κB pathways are significant osteoclast pathways for formation (Takayanagi, 2005; Park et al., 2017). The increased activity of MAPK and NF-κB pathways under inflammatory conditions promotes osteoclast activity and function. Previous studies showed that elevated inflammatory responses and inflammatory factors are related to the development and progression of OP. Furthermore, the immunomodulation of macrophage polarization is a crucial OP mechanism (Singh et al., 2012). M0 macrophages are transformed into proinflammatory macrophages (M1 phenotype) in pathological environments, and proinflammatory chemokines are then released. The proinflammatory microenvironment will activate osteoclast signaling pathways, such as NF-kB, and induce the differentiation of hematopoietic monocytes/macrophages. Meanwhile, M2 macrophages secrete anti-inflammatory factors, promote the formation of an anti-inflammatory microenvironment, and inhibit the differentiation and formation of osteoclasts (Yuan et al., 2017; Bai et al., 2018; Mahon et al., 2018). High levels of proinflammatory factors are closely associated with osteoclast-mediated focal bone resorption (Silverman and Sternberg, 2012). Yang et al. demonstrated that CUR exerted immunomodulatory effects on macrophages by inhibiting inflammatory responses, reducing the release of inflammatory factors, and preventing osteoclast formation by improving Akt/NF-κB/NFATc1 signaling (Yang et al., 2020). Similarly, Kim et al. found that CUR inhibited osteoclastogenesis by upregulating the antioxidant enzyme glutathione peroxidase 4 (GPX4) and attenuating RANKL signaling. The anti-osteoclastogenic effect of CUR appears to be mediated by inhibiting the expression of MAPK and its downstream transcription factor NFATc1 (Kim et al., 2011). Thus, CUR could be helpful in anti-OP by inhibiting osteoclast activity and its mediated bone resorption.

## 4 Effects of curcumin on different animal models of osteoporosis

OP is divided into primary and secondary OP. Primary OP mainly includes postmenopausal OP and senile OP. Postmenopausal OP mainly results in rapid bone loss due to increased osteoclast activity (Maggio et al., 2003). Secondary OP is primarily due to decreased bone mass and bone destruction caused by drugs (e.g., glucocorticoids) or the effects of diseases (e.g., diabetes) (Stein and Shane, 2003). Animal models of OP are suitable tools to investigate prevention and treatment methods. Researchers have established various animal models to determine the therapeutic value of CUR based on the possible pathological mechanisms of OP (Komori, 2015); such models include ovariectomy (OVX) animal model, glucocorticoid OP (GIOP) animal model, diabetic OP (DOP) animal model, and hind limb suspension (HLS) animal model with bone loss due to mechanical unloading. Here, we summarize the available scientific data collected from studies of CUR on several of the animal models mentioned above. This evidence supports CUR‘s beneficial effects in ameliorating different OP types (Table 2).

**TABLE 2:** The preclinical study of curcumin on different rodent models in osteoporosis.

Animals model	Dosage range	Active concentration	Functions	Signaling pathways/Mechanisms	References
OVX model					
*In vivo*, OVX mice model	110 mg/kg for 2 months	10 mg/kg	Stimulates bone formation	EZH2/Wnt/β-catenin signaling pathways	French et al. (2008), Kim et al. (2011), Cho et al. (2012), Hussan et al. (2012), Cho et al. (2013), Jiang et al. (2021)
*In vivo*, OVX rat model	50 mg/kg for 8 or 12 weeks	50 mg/kg; 75 mg/kg; 110 mg/kg	Promotes the increase of trabecular bone density		
	75, 150,750 mg/kg for 6 months				
	10 and 50 mg/kg for 4 or 8 weeks				
*In vivo*, OVX mice model	50 mg/kg for 4 or 8 weeks	50 mg/kg	Inhibits bone resorption	——	French et al. (2008), Kim et al. (2011), Cho et al. (2012), Hussan et al. (2012), Cho et al. (2013), Liang et al. (2020b)
*In vivo*, OVX rat model	200 mg/kg for 4 weeks	75 mg/kg; 110 mg/kg; 200 mg/kg	Promotes the increase of trabecular bone density		
	75, 150 and 750 mg/kg for 6 months				
	110 mg/kg for 2 months				
GIOP model					
*In vivo*, GIOP rat model	100 mg/kg for 2 months	100 mg/kg	Protects bone loss and promotes bone formation by inhibiting apoptosis	Wnt/β-catenin signaling pathway	Chen et al. (2016a), Chen et al. (2016b)
			Promotes the increase of trabecular bone density	Suppresses the formation of apoptosis	
*In vivo*, GIOP mice model	200 mg/kg for 12 weeks	200 mg/kg	Inhibits bone resorption	OPG/RANKL/RANK signaling pathways	Li et al. (2015)
			Promotes the increase of trabecular bone density		
DOP model					
*In vivo*, DOP rat model	100, 110 or 120 mg/kg for 2 or 8 weeks	100 mg/kg	Inhibits bone resorption and prevents bone loss	NF-κB and TGFβ/Smad2/3 signaling pathways	Hie et al. (2009), Liang et al. (2020a), Deng et al. (2021), Fan et al. (2022)
*In vivo*, DOP mice model			Promotes the increase of trabecular bone density		
*In vivo*, T2DOP rat model					
HLS model					
*In vivo*, HLS rat model	110 mg/kg for 6 weeks	110 mg/kg	Prevents bone loss by inhibiting oxidative stress	Inhibits oxidative stress	Xin et al. (2015)
			Promotes the increase of trabecular bone density		

Abbreviations: OVX, ovariectomy; EZH2, enhancer of zeste homolog 2; Wnt, wingless and int-1; GIOP, glucocorticoid osteoporosis; OPG, osteoprotegerin; RANKL, nuclear factor receptor activator kappa B ligand; RANK, nuclear factor kappa B; DOP, diabetic osteoporosis; T2DOP, type 2 diabetic osteoporosis; NF-κB, nuclear transcription factor-κB; Smad2/3, recombinant mothers against decapentaplegic homolog 2/3; HLS, hind-limb suspension.

### 4.1 Curcumin and estrogen-deficient osteoporosis

The OVX model is the most commonly used animal model of postmenopausal OP. Estrogen inhibits osteoclast formation and differentiation by binding to the osteoclast precursor receptor (Birmingham et al., 2012; Li et al., 2012). When estrogen is deficient, it increases osteoclast activity and causes significant bone loss. Therefore, effective prevention of estrogen deficiency-induced increase in osteoclast activity and bone loss has potential for the treatment of OP.

**FIGURE 4:**
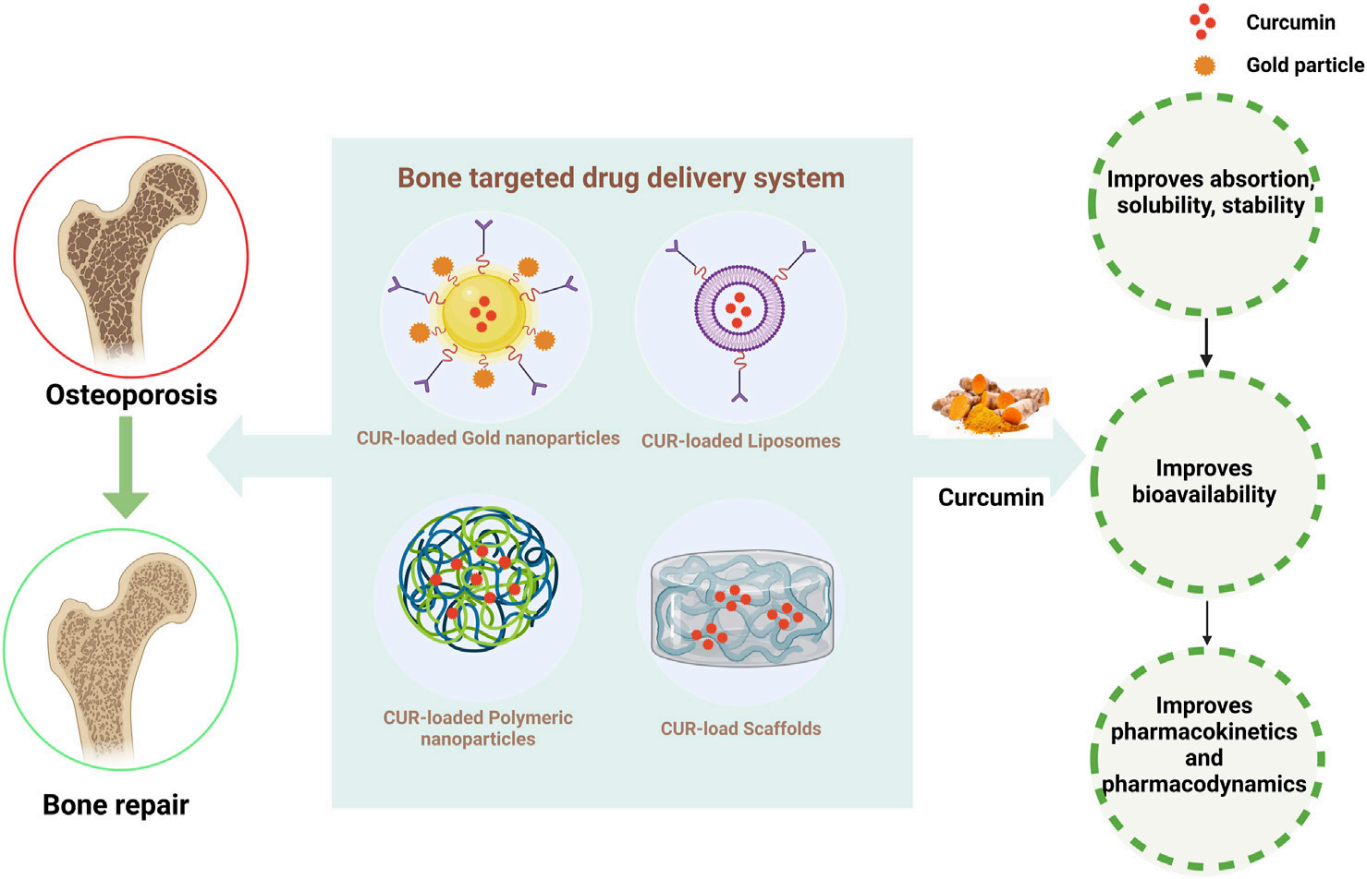
Strategies for improving the pharmacokinetics of CUR. The figure illustrates that curcumin combined with different drug delivery systems can significantly improve the bioavailability and better treat osteoporosis.

CUR has well-documented functional applications and therapeutic potential in treating estrogen-deficient bone loss. OVX-induced bone loss may be associated with elevated ROS activity (Maggio et al., 2003). Kim et al. found that in a mouse model of OVX, continuous treatment with CUR (9.5 μg/g/d) for 8 weeks significantly inhibited OVX-induced bone loss by upregulating the antioxidant enzyme GPX-1 activity and reducing the formation of the bone resorption marker type I collagen C-terminal peptide (CTX-1) (Kim et al., 2011). Liang et al. evaluated the effect of oral CUR (200 mg/kg/d) on bone trabeculae in OVX mice. OVX caused bone loss by promoting the production of CCL3 in bone trabeculae, and the effect was effectively reversed by CUR (Liang Z. et al., 2020). However, Folwarczna et al. found that although continuous dietary CUR (10 mg/kg/d) for 4 weeks slightly improved the morphological parameters of bone tissues damaged by estrogen deficiency, it did not improve bone mineralization or bone strength (Folwarczna et al., 2010). Frank et al. designed three doses of CUR (1.5, 3, and 15 mg/kg) and studied their osteoprotective effects after spinal fusion in OVX rats. Although the results showed increased spinal bone mineral density (BMD) in all three CUR groups compared with the OVX group, the difference was not statistically significant (Cho et al., 2017). This lack of effect occurs possibly because CUR is less bioavailable and has a shorter half-life in animals, so small doses may not be helpful for the prevention or treatment of OP. Increasing the amount of CUR may have protective effects on bone reconstruction Figures 1, 2, 3, 4.

Several studies have shown the beneficial effect of high doses of CUR on bone turnover and the increased bone strength in a mature rat model of OVX-induced postmenopausal OP (French et al., 2008; Cho et al., 2013). Cho et al. measured the spine of OVX SD rats fed with a steady diet of CUR (10 or 50 mg/kg/d) for 4 or 8 weeks. Both doses of CUR attenuated the loss of spinal BMD and improved bone strength in rats. The high-dose group of CUR was significantly superior to the low-dose group in terms of anti-bone resorption and improving bone strength (Cho et al., 2013). Cho et al. also found that combining CUR with alendronate was superior to a single treatment in anti-bone resorption and improved bone mechanical strength (Cho et al., 2012). Considering that CUR has similar efficacy to estrogen, Hussan et al. compared its therapeutic effect (110 mg/kg/d) with that of ephemeris (100 ug/kg/d) for 8 weeks in OVX rats. The reduction in bone trabeculae and bone loss in OVX rats due to estrogen deficiency was reversed by CUR. Moreover, the therapeutic effect was superior to that of Bemelia (Hussan et al., 2012). Jiang et al. conducted a mechanistic study of CUR on estrogen deficiency-induced OP. The high expression of EZH2 and its regulation of bone resorption-related pathways might be involved in the pathogenesis of estrogen deficiency-induced OP (Jing et al., 2016). CUR supplementation (110 mg/kg/d) protected against OVX-induced OP by modulating the enhancer of zeste homolog 2 (EZH2)/Wnt/β-catenin pathway after 12 weeks (Jiang et al., 2021). However, most current *in vivo* studies on CUR in estrogen-deficient OP have been limited to superficial studies, and fewer studies have addressed its molecular mechanisms. In summary, CUR could be a potential candidate for the prevention and treatment of bone loss in postmenopausal OP.

### 4.2 Curcumin and glucocorticoid osteoporosis

GIOP is the most common form of secondary OP (Laurent et al., 2022). Reduced bone formation and increased bone fragility due to osteoblast apoptosis play a vital role in the pathogenesis of GIOP (Weinstein et al., 1998). Glucocorticoids can accelerate bone loss by promoting RANKL activity and osteoclast formation (Yao et al., 2008). Therefore, preventing the apoptosis of osteoblasts or accelerating the apoptosis of osteoclasts would be a promising therapeutic strategy for the treatment of GIOP.

CUR promotes bone formation and is thought to help improve GIOP. In a rat model of Dex-induced GIOP, Chen et al. found that the administration of CUR (100 mg/lg/d) increased the femurBMD, improved the bone trabecular structure in rats, and significantly reversed the Dex-induced apoptosis in femoral osteoblasts. *In vitro* results also showed that CUR protected osteoblasts from Dex-induced apoptosis by inhibiting the expression of pro-apoptotic proteins and upregulating the ERK pathway (Chen et al., 2016a). Furthermore, substantial evidence suggests that miRNAs are involved in the progression of GIOP (Liu et al., 2017). CUR may regulate miRNAs to ameliorate glucocorticoid-induced secondary OP. Li et al. found that CUR supplementation (200 mg/kg/d) improved gastroprotection in GIOP mice by upregulating tibial miR-365 expression and inhibiting the osteoprotegerin (OPG)/RANKL/RANK pathway in GIOP mice, which was considered a potential therapeutic target for the treatment of GIOP (Li et al., 2015). Osteoblast differentiation mediated by the Wnt/β-catenin pathway plays a crucial role in the pathogenesis of GIOP (Karner and Long, 2017). CUR activated the Wnt/β-catenin pathway to promote osteoblast proliferation and differentiation (Tiwari et al., 2014; Tiwari et al., 2016). Chen et al. observed that CUR treatment (100 mg/kg/d) significantly ameliorated the reduced bone mineral density and bone mineral loss in GIOP rats by modulating the Wnt/β-catenin pathway and attenuating osteoblast differentiation dysfunction *in vitro* (Chen et al., 2016a). Moreover, CUR has significantly inhibited osteoclast-mediated bone resorption by stimulating osteoclastic apoptosis (Yamaguchi et al., 2012; Ke et al., 2020). However, CUR has not been reported to play a role in the pathogenesis of GIOP by modulating osteoclast activity. Further studies are needed to confirm whether CUR modulates osteoclasts to exert an ameliorative role in GIOP. However, there have been no reports on CUR’s role in the pathogenesis of GIOP by modulating osteoclast activity, and further studies are still needed to confirm that CUR’s modulation of osteoclasts plays an ameliorative role in GIOP in the future. In conclusion, CUR may be a new therapeutic approach for the treatment of GIOP through osteoprotective effects.

### 4.3 Curcumin and diabetic osteoporosis

In recent years, the adverse effects of diabetes on bone health have received much attention as researchers intensively studied diabetes and its complications (Murray and Coleman, 2019). DOPis a secondary OP characterized by a hyperglycemia-induced reduction in bone mineral density and impaired bone microarchitecture. Chronic hyperglycemia decreased the bone density in diabetic patients (Kurra et al., 2014). However, a specific drug for the clinical treatment of DOP has not been developed yet, and conventional pharmacological therapies, such as bisphosphonates, are ineffective in long-term use and have serious side effects. Therefore, a safer and more effective treatment must be developed.

CUR is a potential treatment for DOP because of its proven ability to protect bone structure and regulate blood lipid levels (Ren et al., 2020; Inchingolo et al., 2022). Evidence suggests that impaired osteogenic differentiation function and angiogenic capacity of BMSCs are essential mechanisms for the development of DOP (Jing et al., 2016; Liao et al., 2016). CUR elicited osteoprotective effects by repairing osteoblasts damaged by high glucose and the osteogenic differentiation of BMSCs (He et al., 2020; Chen et al., 2021). Fan et al. found that CUR pretreatment promoted BMSC-mediated osteogenic differentiation and angiogenic coupling in a high-glucose environment and prevented diabetes-induced bone loss by inhibiting the NF-κB pathway. Further *in vivo* results showed that CUR supplementation (100 mg/kg/d) prevented bone loss and promoted angiogenesis in DOP mice (Fan et al., 2022). In an insulin-dependent diabetic model, the elevated activity of osteoclast markers, such as TRAP, reduced diabetic bone mass (Hie et al., 2007). By contrast, CUR supplementation (120 mg/kg/d) inhibited the increase in bone resorption activity by suppressing TRAP activity and expression in diabetic rats (Hie et al., 2009). In addition, type 2 diabetic OP (T2DOP) has become the most common form of DOP; it can accelerate bone loss and lead to fractures (Leslie et al., 2012). Liang et al. found that CUR treatment (110 mg/kg/d) protected the bone microarchitecture and improved bone mechanical properties in T2DOP rats by modulating the TGFβ/Smad2/3 pathway (Liang Y. et al., 2020). CUR analogs have been extensively studied for their therapeutic effects on DOP. Novel modified CUR, namely, CMC2.24, promoted inflammation regression and reduced hyperglycemia-induced bone loss in diabetic rats (Deng et al., 2021). Hence, CUR and its analogs may alleviate diabetic OP *in vivo* and *in vitro* by exhibiting osteoprotective effects and modulating blood glucose levels.

### 4.4 Curcumin and disuse osteoporosis

The microgravity environment experienced during bed rest, braking, or prolonged spaceflight is a significant risk factor for disuse OP (Rolvien and Amling, 2022). Prolonged exposure to microgravity reduced bone mass due to increased activity of bone resorption and inhibition of bone formation (Pan et al., 2014). Given that experiments during spaceflight are costly and challenging to implement, the HLS rat model can be used to effectively simulate the effects on bone during prolonged bed rest and microgravity environments (Giangregorio and Blimkie, 2002). Exposing rats to HLS accelerated osteoclast differentiation and formation, leading to bone loss. Furthermore, bone loss induced by a microgravity environment may be associated with oxidative stress caused by elevated reactive oxygen activity (Sun et al., 2013). Hence, microgravity-induced oxidative stress is essential in developing disuse bone loss and is expected to be an important target for the treatment of OP.

CUR exerts osteoprotective effects due to its antioxidant properties. For example, in a rat model of HLS, Xin et al. found that CUR treatment (40 mg/kg/d) alleviated HLS-induced oxidative stress and prevented and restored microgravity-induced bone loss. Further *in vitro* results showed that CUR promoted osteogenic differentiation in the microgravity environment and significantly inhibited osteoclastogenesis (Xin et al., 2015). However, studies addressing the direct effects of CUR on HLS-induced oxidative stress are limited; in this regard, future studies are needed to confirm the osteoprotective results of CUR in disuse bone loss. The above studies suggest that CUR has the potential to prevent and treat disuse OP by inhibiting oxidative stress.

## 5 Clinical trial of curcumin for osteoporosis

CUR, derived from natural plants, is one of the most common bioactive supplements used to treat various chronic diseases, such as cardiovascular diseases, diabetes, tumors, and osteoarthritis (Hong et al., 2019; Abu-Taweel et al., 2020; Pourbagher-Shahri et al., 2021; Yang et al., 2022). *In vivo* and *in vitro* studies have shown that the beneficial effects of CUR on bone are primarily related to improved bone density and mechanical properties, indicating the possible use of CUR to prevent and treat bone diseases (Barik et al., 2020). However, studies on CUR in patients with OP are relatively limited, but all of them reported excellent protective effect.

CUR supplementation significantly improves BMD and bone loss in postmenopausal patients with OP. In a randomized, double-anonymized study of 60 postmenopausal women conducted for 12 months, the combination of CUR (110 mg/d) and alendronate (5 mg/d) significantly improved the BMD status and bone turnover markers in postmenopausal patients with OP, and both were superior to monotherapy. Hence, combining CUR and alendronate may be an effective option for the prevention and treatment of postmenopausal OP (Khanizadeh et al., 2018). In another randomized, triple-blind study of 120 postmenopausal women over 6 months, Khanizadeh et al. found that nanomicellar CUR combined with black seed oil significantly reduced the expression of serum bone turnover markers, namely, ALP, osteocalcin, and osteopontin, and the risk of OP in postmenopausal women (Kheiridoost et al., 2022). This study is the first to apply nanomicellar CUR in a clinical trial, and the process effectively addressed the limitation with regard to the bioactivity of CUR due to its low bioavailability. In another 24-week study of 57 healthy low-density subjects, Riva et al. evaluated the efficacy and safety of a high-dose oral formulation of CUR (1,000 mg/d) in subjects with low bone density. The subjects safely tolerated the CUR treatment and improved BMD (Riva et al., 2017). Furthermore, OP is one of the most common complications in spinal cord injury and has no effective treatment available (Gifre et al., 2014). However, in a 6-month study, Hatefi et al. found that CUR supplementation (110 mg/kg) improved the BMD parameters and reduced the bone turnover biomarker levels in patients with spinal cord injury, thereby inhibiting OP progression (Hatefi et al., 2018). As an active ingredient in natural medicine, CUR has osteoprotective effects on postmenopausal patients with OP. However, the limitations of CUR application in clinical trials are due to its unstable metabolism and low bioavailability in patients. Current strategies to improve CUR’‘s bioavailability mainly include changing CUR’‘s mode of administration or co-administration (Tabanelli et al., 2021). As an active ingredient in natural medicine, the above suggests that CUR has a favorable osteoprotective effect in postmenopausal OP patients. In a word, further studies are needed to determine the specific mechanism of action of CUR in patients with OP.

## 6 Strategy to improve pharmacokinetics of CUR

Increasing lines of evidence indicate that CUR, derived from natural plants, can prevent and treat bone-related diseases due to its biological activities, such as low toxicity and a wide range of molecular targets (Pandey et al., 2018; Inchingolo et al., 2022). However, limitations of CUR in therapy, such as poor stability, low solubility, poor bioavailability, and rapid metabolism, have limited its use in clinical trials (Devassy et al., 2015; de Souza Ferreira and Bruschi, 2019). Therefore, current research focuses on developing well-targeted, stable, slow-release, and low-toxicity bone-targeted drug delivery systems (Chen et al., 2022). Researchers have proposed various nanomaterials, such as nanoparticles, liposomes, and scaffolds, as carriers for bone-targeted drug delivery. Nanomaterials have unique structures with adjustable shape, size, and surface properties and have a crucial effect on drug loading and release, cellular uptake, and blood circulation metabolism (Singh et al., 2019). Nanotechnology ameliorates the problems mentioned above with CUR. The appropriate use of natural compounds in nanotechnology-based bone-targeted drug delivery strategies is a promising therapeutic strategy to improve bone metabolic diseases Figure 4, Table 3.

**TABLE 3:** New applications of curcumin nanoformulations in osteoporosis treatment.

Type of nanoformulations	Experimental model (Animals/Cells)	Dosage range	Active concentration	Functions	Signaling pathways/Mechanisms	References
CUR/ALN nanoparticles coated with HA	*In vitro*, MC3T3-E1 cells	0.5–10 μM	0.5 μM	Promotes osteoblasts differentiation, proliferation and mineralization	Keap 1/Nrf2/HO-1 signaling pathways	Jebahi et al. (2015), Dong et al. (2018), Li and Zhang (2018)
CUR-encapsulated chitosan-bioglass	*In vivo*, Surgery-induced femoral condyle defect Wistar rats	25 mg/kg for 4 weeks	25 mg/kg	Stimulates bone formation		
CUR-loaded PLGA microspheres	*In vitro* and *vivo,* HG-induced BMSCs/T2OP rat model					
CUR-loaded CD gold nanoparticles	*In vitro* and *vivo*, BMMs and osteoclasts induced by RANKL/OVX mice model	0.5–10 μM	0.5 μM	Inhibits osteoclasts differentiation and formation	MAPK and OPG/RANK/RANKLsignaling pathways	Heo et al. (2014), Partoazar and Goudarzi (2022)
CUR-loaded PSL liposomes	*In vivo*, GIOP rat model	25 mg/kg for 3 weeks	25 mg/kg	Inhibits bone resorption and prevents bone loss		

Abbreviations: CUR, curcumin; ALN, alendronate; Keap 1,kelch-like ECH-associated protein 1; Nrf2, nuclear factor-like 2; HO-1, Heme oxygenase-1; PLGA, polylactic acid-hydroxyacetic acid copolymer; BMSCs, bone marrow stromal cells; T2DOP, type 2 diabetic osteoporosis; CD,β-cyclodextrin; PSL, phosphatidylserine liposomes; BMMs, bone marrow-derived macrophages; RANKL, nuclear factor receptor activator kappa B ligand; OVX, ovariectomy; GIOP, glucocorticoid osteoporosis; MAPK, mitogen-activated protein kinase pass; OPG, osteoprotegerin; RNAK, nuclear factor kappa B; RANKL, nuclear factor receptor activator kappa B ligand.

Recent studies have discussed the protective effect of bone-targeted drug delivery strategies for regulating bone reconstruction by delivering CUR through nanoparticles and liposomes. Dong et al. constructed an *in vitro* nanoparticle delivery system loaded with alendronate and CUR (HA-ALN/CUR) as a novel OP treatment. HA-ALN/CUR showed enhanced ability to stimulate bone formation by upregulating bone formation markers, such as bone morphogenetic protein-2 (BMP-2), Runx2, and OCN, in MC3T3-E1 cells, ultimately promoting bone regeneration (Dong et al., 2018). Gold nanoparticles (GNPs) are the most suitable bioactive material for bone tissue engineering applications to prevent OP by inhibiting the formation of osteoclasts (Sul et al., 2010). Heo et al. developed a device that combines CUR with cyclodextrin loaded on GNPs (CUR-CGNPs). CUR-CGNPs prevented the differentiation of BMMs into osteoclasts by inhibiting the expression of RANKL-induced osteoclastogenic transcription factors c-Fos and NFAT1 and activating the downstream related pathway MAPK. In in *vivo* models, CUR-CGNPs also improved the bone mineral density and microarchitectural bone parameters in the OVX mouse model; hence, CUR-CGNPs may be potential novel therapeutic agents for the prevention and treatment of OP (Heo et al., 2014). In a GIOP rat model, Partoazar et al. treated phosphatidylserine liposomes containing CUR (PSLs-CUR) and observed an excellent improvement in bone density and bone mechanical properties possibly through the modulation of the OPG/RANK/RANKL pathway (Partoazar and Goudarzi, 2022).

Many patients who underwent radiotherapy develop OP and skin damage problems. The elevated free radicals and consequent increase in oxidative stress caused by ionizing radiation may be associated with OP and skin damages (Rana et al., 2012; Mamalis et al., 2014). Nanoparticle carriers based on polymers, such as chitosan and polylactic acid-hydroxyacetic acid copolymer (PLGA), are widely used for drug delivery and bone repair given their drug stability, biodegradability, and biocompatibility (Chen et al., 2022). Jebahi et al. designed chitosan complexes loaded with CUR and bioactive glass (BG) to significantly improve the bioavailability and antioxidant properties of CUR, promote skin healing, and increase the bone density in rats after radiation by scavenging free radicals and reducing oxidative stress (Jebahi et al., 2015). Li et al. found that PLGA microspheres loaded with CUR significantly ameliorated the harmful effects of proliferation, migration, and osteogenic differentiation of spinal cord BMSCs in a high-glucose state and promoted bone formation in diabetic rats by modulating the Kelch-like ECH-associated protein 1(Keap1)/Nrf2/Ho-1 signaling pathway (Li and Zhang, 2018). Thus, combining CUR and nanoformulations may be a unique biotherapeutic tool for preventing and treating OP.

## 7 Summaries and perspectives

In conclusion, as a natural compound, CUR may play a key role in preventing and treating OP. Over the past few decades, CUR has received extensive attention from researchers because of its remarkable bone-protective effects and promising therapeutic potential. Preclinical and clinical studies showed that CUR acts on osteoblasts and osteoclasts through various mechanisms to promote osteogenic differentiation and inhibit osteoclastogenesis, thereby exerting beneficial effects on bone formation. Evidence from various animal models of OP confirms the therapeutic effect of CUR supplementation on bone. CUR attenuates oxidative stress, apoptosis, and inflammatory activation in OP. CUR is considered a safe, inexpensive, and novel therapeutic agent with few side effects and thus could be a promising alternative therapy for the prevention and treatment of OP.

However, despite the breakthroughs in recent years regarding the therapeutic activity of CUR in OP, many issues need to be urgently addressed. First, based on the evidence available from *in vitro* studies, CUR offers a promising natural therapeutic agent for OP by acting on osteoblasts and osteoclasts. However, it was impossible to determine *in vitro* whether the effects of CUR on osteoblasts and osteoclasts translate into bone tissue. Furthermore, studies on the biological activity of CUR still need to be completed. The therapeutic limitations of CUR, such as its low solubility, bioavailability insertion, and rapid metabolism, have limited its application in clinical trials. Therefore, researchers have proposed nanotechnology-based strategies such as micelles, liposomes, and binding polymers to improve and enhance the application of CUR in preclinical and clinical trials. In addition, modifying the chemical structure of CUR or combining it with other compounds to synthesize multi-targeted CUR analogs can improve the bioavailability and efficacy of OP. Thirdly, animal model studies demonstrated the therapeutic value of CUR for bone. CUR has significant efficacy as a potent anti-inflammatory agent in various diseases, but studies on the anti-inflammatory effects of CUR in OP are limited. Further studies are needed to confirm the therapeutic role of CUR as an anti-inflammatory agent in OP. Finally, some studies have suggested that phytochemicals such as CUR may be pan-analytical interfering compounds (PAINS). Such compounds are believed to give false positive test results by interfering with various reactions in the assay such as chemical polymerization, fluorescence interactions, and the presence of active Michael receptors, rather than through specific target/compound interactions. Not only that, the results are also interfered with by the experimental conditions such as the purity of the phytochemicals extracted (Baell, 2016). Due to the wide range of pharmacological activities of these phytochemicals in various diseases thus considered as a panacea by most researchers. However, this ineffective cure-all (IMPS) may result in the wastage of resources in drug development and thus requires deeper exploration in terms of drug extraction and experimental conditions to minimize the impact of false positive results on the trials (Bisson et al., 2016). In conclusion, CUR may be able to treat OP by attenuating apoptosis, oxidative stress, and inflammatory responses in order to avoid becoming an IMPS due to its pharmacological effects as a false-positive, but the specific molecular mechanisms regarding CUR’‘s effects on different types of OP and its function in con-osteoporosis still need to be further investigated, and further experimental demonstration is needed based on the existing foundation. There are still limitations in our review, and the obtained results have the risk of false positives. However, in developing natural products based on the potential to target OP, our review is still based on summarizing and critically evaluating the existing evidence to provide reference value for further research and drug development. In conclusion, we expect more researchers and experts to conduct systematic experimental designs and analyses in the future, leading to an effective evaluation system and enabling phytochemicals to benefit OP patients more effectively. Results will provide clinical implications for the prevention and treatment of OP.
